# Effect of Sharp Diameter Geometrical Modulation on the Magnetization Reversal of Bi-Segmented FeNi Nanowires

**DOI:** 10.3390/nano8080595

**Published:** 2018-08-05

**Authors:** Miguel Méndez, Víctor Vega, Silvia González, Rafael Caballero-Flores, Javier García, Víctor M. Prida

**Affiliations:** 1 Departamento de Física, Universidad de Oviedo, C/Federico Garcia Lorca 18, 33007-Oviedo, Asturias, Spain; miguel.mendez82@gmail.com (M.M.); gonzalezgana@uniovi.es (S.G.); rafaelcaballero@uniovi.es (R.C.-F.); garciafjavier@uniovi.es (J.G.); 2 Laboratorio Membranas Nanoporosas, Servicios Científico-Técnicos, Universidad de Oviedo, Campus El Cristo s/n, 33006-Oviedo, Asturias, Spain; vegavictor@uniovi.es

**Keywords:** anodization, atomic layer deposition, diameter modulated nanowire, micromagnetic simulation, ferromagnetic nanowire, MOKE, domain wall, magnetization reversal, Barkhausen jump

## Abstract

Controlling functional properties of matter and combining them for engineering a functional device is, nowadays, a common direction of the scientific community. For instance, heterogeneous magnetic nanostructures can make use of different types of geometrical and compositional modulations to achieve the control of the magnetization reversal along with the nano-entities and, thus, enable the fabrication of spintronic, magnetic data storage, and sensing devices, among others. In this work, diameter-modulated FeNi nanowires are fabricated paying special effort to obtain sharp transition regions between two segments of different diameters (from about 450 nm to 120 nm), enabling precise control over the magnetic behavior of the sample. Micromagnetic simulations performed on single bi-segmented nanowires predict a double step magnetization reversal where the wide segment magnetization switches near 16 kA/m through a vortex domain wall, while at 40 kA/m the magnetization of the narrow segment is reversed through a corkscrew-like mechanism. Finally, these results are confirmed with magneto-optic Kerr effect measurements at the transition of isolated bi-segmented nanowires. Furthermore, macroscopic vibrating sample magnetometry is used to demonstrate that the magnetic decoupling of nanowire segments is the main phenomenon occurring over the entire fabricated nanowires.

## 1. Introduction

Nanoporous anodic alumina templates electrochemically engineered by anodization [1], allow the obtaining of elaborated and reproducible 3D arrangements of self-ordered nanopores with precise control on their lattice parameters, such as the pore diameter, interpore distance, pore length, and geometry. The peculiar features of the nanoporous alumina membranes make them outstanding patterned platforms in a great variety of applications, such as for energy conversion and wastewater treatment, information storage, microfluidics, orthopedic prosthetics, dental and coronary stents, cell culture scaffolds, immunoisolation devices, biomolecular filtration, and drug delivery, among others [2,3,4,5,6,7,8,9,10,11,12,13]. 

Three-dimensional (3D) arrays of nanostructured materials, such as dots, nanowires, nanotubes, nanofibers, or antidots, exhibit distinctive physical properties with respect to their bulk counterparts due to their specific low dimensions at the nanoscale, which allow them to play a very important role in the fields of nanoscience and nanotechnology [14,15,16,17,18]. Metallic, magnetic, and semiconducting nanowires (NWs) are, nowadays, the focus of a great deal of attention among scientists coming from different research fields, due to the peculiar physico-chemical features exhibited by these materials with nanometer-sized dimensions and specific tailored geometries. Therefore, wire-shaped nanomaterials have become key elements in many modern technological approaches, including high-density magnetic data storage, energy conversion and harvesting, catalysis, sensing, photonics, and many others, where the size and shape of the nanoelements become essential to determine the unique magnetoelectronic properties exhibited by these systems [19,20,21].

Nanowire-based thermoelectric structures have been investigated in order to achieve improved thermoelectric performance of the future generation of efficient thermoelectric devices, because the downsizing to nanoscale dimensions of these materials allows for increasing their surface to volume ratio in a controlled way and, thus, increase the diffusive phonon scattering, which should revert into an increase of the figure of merit [22].

At the same time, ferromagnetic nanowires grown by template-assisted electrochemical deposition inside the hexagonally self-ordered pores of the alumina membranes have been proposed for exploiting the third dimension of space in 3D magnetic data storage material systems based on the so-called ‘racetrack’ model [23]. The parallel arrays of vertically-aligned nanowires can be used to increase the areal storage density by several orders of magnitude despite the lower limit on bit size, in order to store the magnetic information in the form of sectors of wires having the same ferromagnetic alignment. Such a system relies on the shape anisotropy of each magnetic nanowire as the stabilizing mechanism, avoiding the spontaneous magnetization reversal, but such a stabilization mechanism weakens with the decreasing size of the nanowires [24]. Improving magnetic data storage has been foreseen to be possible by introducing segmented ferromagnetic nanowires with geometrical or compositional modulations that produce a self-stabilization mechanism [25]. For some particular geometrical configurations it allows making the magnetostatic interaction among different segments of the nanowire the most important one to stabilize the system, leading to more durable stored information by avoiding the aging phenomenon due to the spontaneous magnetization reversal of individual nanowires, which causes the stored magnetic data to be gradually lost [26].

Multisegmented magnetic nanowires produced by varying both the chemical composition of each segment [27,28,29,30,31,32], or more recently by properly tuning the geometrical modulation in the diameter of each nanowire segment [33,34], have been proposed as novel 3D systems of magnetic multibit memories and logical devices. This peculiar assembling of building blocks made of consecutive segments with modulated composition and/or diameter for each nanowire, allows for the magnetization confinement in each nanowire segment, giving rise to arrays of nanowires with a magnetic multi-domain structure along the wire length, where the interface layer at the modulation can act as a pinning center for magnetic domain wall displacement [35].

In this work, we report on the synthesis, morphology, and microstructure of bi-segmented FeNi alloyed nanowires geometrically modulated in diameter, together with the analysis of their magnetic properties by the vibrating sample magnetometry (VSM) technique for the bulk nanowires array, or well in the isolated nanowires by the magneto-optical Kerr effect, after releasing them from the patterned alumina substrate. The soft magnetic character exhibited by these ferromagnetic nanowires made of nickel-iron alloy, also known as Permalloy, which is widely employed as a magnetic shield, for instance [36,37,38], is found to come from their magnetocrystalline anisotropy, which is much weaker than the shape anisotropy and, therefore the magnetostatic interactions govern the magnetic behaviour of the material. The diameter-modulated FeNi nanowires were fabricated paying special emphasis to obtain a sharp transition region between the two nanowire segments with different diameters, ranging from 450 nm of the thicker segment and down to 120 nm for the thinner one, thus enabling the precise control over the magnetic behaviour of the sample. The influence of the sharp diameter modulation on the magnetization reversal process of these ferromagnetic nanowires has also been examined by micromagnetic simulations performed with the mumax3 package, in order to determine the main magnetic domain wall propagation mechanism of the magnetization reversal for each segment of the diameter modulated FeNi nanowire. A comparison between experimental measurements and micromagnetic simulations demonstrate the good agreement achieved among the obtained results. These geometrically diameter-modulated ferromagnetic nanowires studied here can be considered as novel magnetic multidomain systems for ultrahigh-density data storage applications, in a similar way to racetrack memory devices.

## 2. Materials and Methods

### 2.1. Tailor-Made Nanoporous Alumina Templates with Geometrically Tunable Pore Diameters

Patterned templates made of nanoporous alumina membranes with hexagonally self-ordered and diameter-modulated nanopores were synthesized by a novel multistage procedure consisting in the combination of electrochemical anodization, atomic layer deposition (ALD), and pore widening steps, performed on high-purity aluminum foils as the starting substrates, in a similar way to that recently reported by Prida et al. [39], which has been adapted in order to obtain wider pore diameters. 

**Figure 1:**
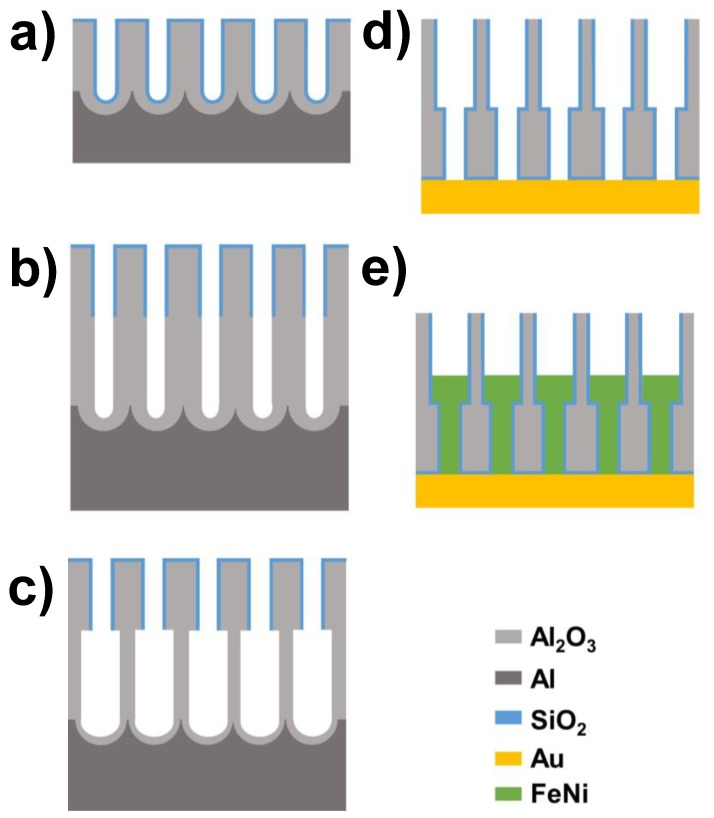
Fabrication flowchart of diameter-modulated FeNi alloy nanowire arrays by template-assisted deposition in tailored geometrically modulated nanoporous alumina membranes. (**a**) Second anodization and SiO_2_ deposition. (**b**) Third anodization. (**c**) Pore widening. (**d**) Al substrate and barrier layer removal, ALD coating and gold contact deposition. (**e**) Electrodeposition growth of bi-segmented NiFe alloy nanowires.

Before the first anodization process, the high-purity Al foils (Al 99.999%, Goodfellow, Huntingdon, UK), employed as starting material substrates, were cleaned by sonication in ethanol and isopropanol, and then electropolished by applying an anodic voltage of 20 V versus a platinum counter-electrode in a mixture of perchloric acid and ethanol (1:3 vol.%) at 5 °C. After the sample’s surface roughness is reduced to obtain a mirror-like finishing, the polished Al substrates were anodized in 1 wt.% orthophosphoric acid electrolyte for 24 h at 1 °C and under an applied voltage of 194.5 V, in order to achieve a highly ordered, hexagonally arranged nanoporous structure [40,41]. This firstly grown anodic alumina oxide layer was removed by selective chemical etching in 0.2 M CrO_3_ and 0.6 M H_3_PO_4_ aqueous solution heated at 30–40 °C for 48 h. The samples were then re-anodized for four hours under the same conditions, thus giving rise to the first stage of the patterned template consisting of the narrower segment of the diameter modulated alumina nanopores. Then, a 20 nm thin SiO_2_ nanopores coating was deposited by ALD technique [42], in order to define and fix the diameter of the nanopore’s narrow segment, by depositing the conformal SiO_2_ layer that exhibits enhanced resistance to chemical etching processes (Figure 1a), as long as the layer thickness is greater than 4–5 nm [43,44]. Non-SiO_2_ coated layer prolongation of previously grown nanopores is achieved by re-anodizing the samples under the same conditions for 48 h (Figure 1b). The absence of such coating in that nanopores segment, makes them more sensitive to further chemical etching, thus enabling to widen the unprotected segment with respect the coated one (Figure 1c). Immersing the anodized samples in 10% H_3_PO_4_ solution for 3.5 h resulted in a sharp geometrical modulation of the pore diameter at the interface between the two nanopore segments, from the initial 150 nm of the SiO_2_-coated segment to about 450 nm for the non-coated one. After the anodization and pore widening steps, the remaining Al substrate was dissolved in CuCl_2_ and HCl solution, and the alumina barrier layer occluding the bottom of the pores was etched away in 10 wt.% H_3_PO_4_ solution. Finally, the alumina membrane was exposed again to a new ALD deposition step of a 9-nm thick SiO_2_ coating, creating a passivation layer covering the inner surface of the alumina nanopores. Therefore, such final coating will also cover the whole surface of the nanowires being electrodeposited inside the nanopores, preventing oxidation and corrosion, and additionally enhancing their mechanical stability. However, prior nanowires electrodeposition, a seed conductive gold layer was deposited in the upper side of the nanoporous alumina membranes by sputtering and further electrodeposition steps, serving as a working electrode (Figure 1d).

### 2.2. Template Assisted Electrodeposition of FeNi Alloy Nanowires

The deposition of the FeNi alloy nanowires was performed by using the diameter-modulated nanoporous alumina membranes, employed as templates, in a three-electrode setup suited with an Ag/AgCl reference electrode and Pt counter-electrode. An aqueous solution of NiSO_4_ (90 g/L), FeSO_4_ (14 g/L), ascorbic acid (1 g/L), and boric acid (25 g/L) was employed as the electrolyte [45]. A DC voltage of −1.4 V vs. the reference electrode was applied to the working electrode, resulting in the complete filling of the narrow segment and the partial filling of the wider one in a controlled manner (Figure 1e). By controlling the deposition time in the range between 480 and 900 s it was possible to synthesize two different FeNi diameter-modulated nanowire array samples with varying lengths of the wide segment. First of all, we performed the study on bi-segmented nanowires whose different segments had the same length ensuring the magnetic bistability in both segments. However, due to the larger volume of the wider segment, and thus the higher contribution to the net magnetic moment of the sample, it would hide the signal coming from the narrow segment during the magnetic characterization. For that reason, we reduced the length of wider segment and, therefore, its volume, in order to diminish its contribution to the overall magnetic signal of whole sample, to be around three times larger than the one of the narrow segment, but still ensuring a well-defined shape magnetic anisotropy. The different types of samples fabricated in this study are hereafter labelled referring to the wide modulations length. In sample labelled as WM2, the length of this segment has been selected to be about 2 µm, whereas for sample WM9 it takes values of around 9 µm. For both WM2 and WM9, the length of the narrow segment was fixed to 9 µm. Similar electrodeposition conditions using nanoporous alumina templates with a homogeneous pore size were also employed for the growing of FeNi alloyed nanowire arrays with single wire diameters corresponding to both types, the narrow (N9) and wider diameters (W2 and W9), respectively, of bi-segmented nanowires, in order to compare their magnetic behaviour.

### 2.3. Characterization of the Morphological, Magnetic and Magneto-Optic Properties of Samples

Scanning electron microscopy (SEM, JEOL 5600, Akishima, Tokyo, Japan) equipped with an energy dispersive X-ray (EDX) microanalysis system (INCA, Oxford Instruments, Abingdon, UK) was employed to characterize the morphology and chemical composition of the bi-segmented FeNi nanowires embedded in the nanoporous alumina templates. Morphology and geometrical parameters of freestanding single nanowires were verified by transmission electron microscopy (TEM, JEOL-2000-EXII, Akishima, Tokyo, Japan).

The longitudinal Kerr effect was employed to measure the hysteresis loops of single nanowires along their main axis by means of a NanoMOKE3 (Durham Magneto Optics Ltd., Durham, UK), suited with a quadrupole magnet that reaches fields up to ±95 kA/m. Magnetic measurements of FeNi nanowire arrays were performed at room temperature (RT) in a vibrating sample magnetometer (VSM, Versalab, Quantum Design Inc. San Diego, CA, USA), under applied magnetic fields up to ±3 T. 

### 2.4. Micromagnetic Simulations of Diameter Modulated Single Nanowires

Micromagnetic simulations of single magnetic nanowires have been performed using the GPU-accelerated Mumax^3^ software (written and maintained by Arne Vansteenkiste, DyNaMat group of Prof. Van Waeyenberge (http://dynamat.ugent.be), Ghent University, Belgium), which is based on finite-difference discretization of space using a 2D or 3D grid of orthorhombic cells treating volumetric quantities, like magnetization and effective field at the centre of each cell [46]. A dual NVIDIA GeForce Titan Xp is being used as the hardware for the simulations. This software uses constructive solid geometry to define the shape and composition of the different parts of the magnetic material object of study. This software package allows the calculation of the temporal evolution of the reduced magnetization based on the modified Landau-Lifshitz-Gilbert-Slonczewski (LLGS) equation of movement for the magnetization, $\vec{m}\left(\vec{r},t\right)$. Its time derivative has to consider three contributions: Landau-Lifshitz torque, Zhang-Li spin-transfer torque and Slonczewski spin-transfer torque. A finite difference discretization allows the magnetostatic field to be evaluated as a discrete convolution of the magnetization [46]. Paraview software has been employed to obtain the 3D representation of the magnetic moment configuration of the studied nanowires. Geometries designed for the micromagnetic simulation of diameter modulated nanowires consist of two segments with different diameters for the narrow and wide segment (120 nm and 450 nm, respectively). The length of both segments was kept fixed at a value of 9 µm, thus mimicking the geometry of the experimental WM9 nanowire. The material of choice for the micromagnetic simulations was Permalloy (Py) since it fits best with the experimentally-obtained composition of nanowires. Material parameters for Py were assumed to be 860 × 10^3^ A/m for the saturation magnetization (M_S_), 13 × 10^−^^12^ J/m for the exchange stiffness constant (A_exch_), and a value of the Gilbert damping constant (α) of 0.0055. Reported values for the exchange length (*l_exch_*) in Py are in the range of 5.3–5.7 nm [46,47], while a value of 5.69 nm is typically accepted and will be adopted in this work. Therefore, the cell size has been selected trying to maintain a relation between cell and grid sizes below 75% of *l_exch_*. However, in order to maintain a reasonable computation time, it has been necessary to rescale the dimensions of the nanowires by a factor 1:3, with respect to the originally-mentioned geometry [48]. 

## 3. Results

### 3.1. Morphological and Compositional Characterization of Bi-Segmented FeNi Nanowires

**Figure 2:**
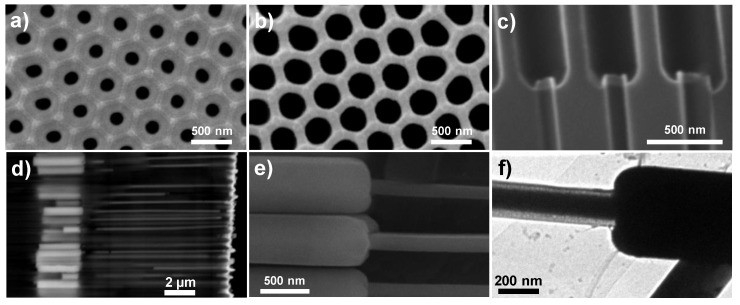
Morphological characterization of diameter modulated nanoporous alumina membranes and FeNi nanowires. (**a** and **b**) SEM images of a nanoporous alumina membrane obtained at the narrow (**a**) and wide (**b**) pore diameter surfaces. (**c**) Cross-section SEM image of the anodic alumina template showing the sharp modulation in the pore diameter of each segment, at the interface. (**d**) Cross-section SEM image of sample WM2 with diameter modulated FeNi nanowires embedded in the alumina template. (**e**) High-magnification SEM image taken at the modulation interface of the FeNi nanowires embedded into the nanoporous alumina templates. (**f**) TEM image of a single and freestanding bi-segmented FeNi nanowire, showing the sharp diameter modulation between the two nanowire segments and the thinner SiO_2_ cover layer.

Figure 2 summarizes the results obtained from the morphological characterization of diameter modulated nanoporous alumina templates and the corresponding nanowires grown by the template-assisted electrochemical deposition technique. Figure 2a,b correspond to SEM images of the narrow and wide pore surfaces of the modulated membranes. In both images the hexagonal highly ordered pore arrangement with lattice parameter *D* ≈ 480 nm, can be observed. Furthermore, the large difference in average pore diameter (*d)* between the narrow and wide pores becomes clear. As a consequence of the pore diameter variation, the porosity of the nanoporous template, $P\;=d^{2}\pi /\left(2D^{2}\sqrt{3}\right)$ takes values of 10% and 50% in the narrow and wide pore surfaces, respectively [40]. The sharp modulation in the nanopores’ diameter (from nearly 450 nm to about 120 nm) is even more evidenced in the image shown in Figure 2c, which corresponds to a SEM cross-section view of a mechanically broken membrane. By the electrochemical deposition method, it has been possible to fill these diameter-modulated pores of the alumina template from the narrow towards the wide segment, thus resulting in diameter-modulated FeNi nanowire arrays, as depicted in the SEM images of Figure 2d,e. The compositional contrast obtained in the SEM, by employing a backscattered electron detector, allows distinguishing the wide and narrow nanowire segments, as well as the filling degree of the nanoporous templates. Figure 2f shows a TEM image obtained in a freestanding diameter-modulated FeNi nanowire. Again, the sharp diameter variation at the modulation is confirmed from this image. Furthermore, the protective SiO_2_ cover layer of the nanowires can be clearly identified in the narrow segment, due to the lower atomic density of this oxide in comparison to the metallic core of the nanowires.

**Figure 3:**
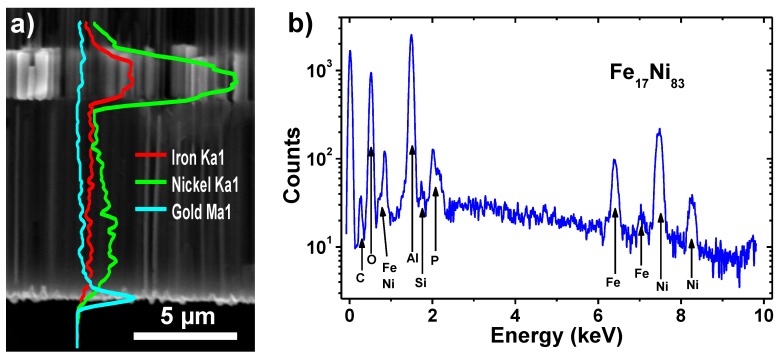
(**a**) EDX compositional profile line-scan performed on a cross-section SEM image of FeNi diameter modulated nanowires; and (**b**) the EDX spectrum indicating the elemental composition of the WM2 sample shown in (**a**).

The distribution of the alloyed elements in the nanowires has been studied by EDX composition profile line scans, as displayed in Figure 3a for sample WM2. In the image, a large increase in the signal arising from both Fe and Ni metals can be observed for the wide segment of the nanowires, in comparison to the narrow one, due to the higher amount of metallic material. Nevertheless, the Fe/Ni ratio keeps approximately constant for all the length of the nanowires, thus indicating their uniform composition. At the bottom of the image a thin gold layer can be found, which corresponds to the gold seed layer employed as a contact for the electroplating steps. The EDX spectrum shown in Figure 3b indicates a Fe_17_Ni_83_ average composition for the magnetic nanowires. Other elements found in the spectrum, such as C, O, Al, P, and Si can be ascribed to the nanoporous alumina template and the ALD deposited SiO_2_ protective coating.

### 3.2. Micromagnetic Simulations of Single Bi-Segmented FeNi Nanowires 

**Figure 4:**
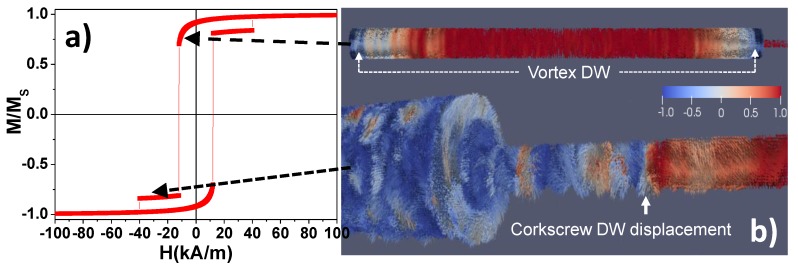
(**a**) Simulated hysteresis loop of sample WM9 showing a magnetization reversal with two Barkhausen jumps. (**b**) Micromagnetic simulation of the magnetic domain structure during the magnetization reversal of a bi-segmented diameter modulated FeNi nanowire at applied fields around 5.6 kA/m, showing the nucleation of two vortex DW at both ends of the wider segment that are propagating to the centre of the segment (**upper**); and the magnetic domain structure of the bi-segmented nanowire at applied fields around 38 kA/m, where the magnetic DW displacement for the magnetization reversal along the thinner segment of the FeNi nanowire, occurs via *corkscrew* movement (**lower**).

Micromagnetic simulations results from sample WM9 are summarized in Figure 4. The hysteresis loop, shown in Figure 4a, clearly presents two separate Barkhausen jumps indicating a two-step magnetization reversal that can be associated with the thick segment first (around 16 kA/m) and the narrow segment afterwards (around 40 kA/m). More precisely, starting from saturation magnetization and reverting the applied magnetic field, a coherent rotation process of the magnetization can be observed in the hysteresis loop at the beginning of the magnetization reversal. This effect can be associated with the nucleation of two vortex domain walls (VDW) at each end of the wide segments as it is deduced from Figure 4b (upper panel) as well as from the reported literature [49,50]. Further reverting the magnetic field, these domain walls move towards the centre of the segment and collapse producing the first Barkhausen jump of the hysteresis loop. Before the complete reversal of the wide segment, a vortex domain wall is injected into the narrow segment with not enough energy to be propagated along the entire length of the narrow segment. The displacement of such VDW along the end of the narrow segments produces the monotonic change of the magnetization between the two Barkhausen jumps of the hysteresis loop. In order to achieve sufficient energy to provoke a majority magnetization reversal of the narrow segment, the applied magnetic field must be further reversed to, in fact, produce the corkscrew DWs movement [51,52], which propagates, afterwards, along the narrow segment (down panel of Figure 4c) and starts its magnetization switching, which is responsible for the second Barkhausen jump in the hysteresis loop, resulting in several nucleation nodes.

### 3.3. Magnetic Characterization of Arrays and Single Bi-Segmented FeNi Nanowires

**Figure 5:**
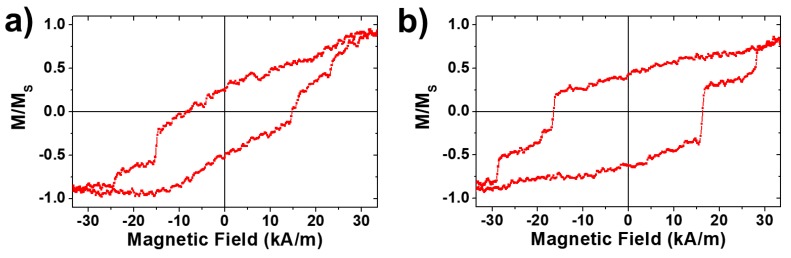
MOKE hysteresis loops of single, isolated and bi-segmented FeNi nanowires with diameter modulation, measured at the narrow/wide segments interface. (**a**) corresponds to sample WM9, while (**b**) was obtained for sample WM2.

With the aim of validating the micromagnetic simulations performed in this work, the magnetization reversal of isolated nanowires has been characterized by means of magneto-optical Kerr effect (MOKE) magnetometry. In order to obtain freestanding single nanowires, the nanoporous alumina template containing the bi-segmented nanowire arrays was dissolved in a 0.2 M CrO_3_ and 0.6 M H_3_PO_4_ solution. The released nanowires were then dispersed and washed in water and ethanol several times. A drop of ethanol containing the suspended nanowires was deposited onto a silicon wafer located in between two permanent magnets, in order to have all the nanowires aligned in parallel along a particular direction of the Si substrate. Such alignment ensures the application of the magnetic field along the long axis of the nanowire during the MOKE measurements. As proof of two-step magnetization reversal, the MOKE hysteresis loop in Figure 5a shows the same qualitative behaviour as the micromagnetic simulations with a small variation on the absolute values of the two Barkhausen jumps. Such variation comes from the fact that experimental nanowires grown by a large scale method typically show a dispersion of their magnetic properties due to small variations in length, diameter, or composition. Furthermore, such variations seem to have a stronger impact on the narrow nanowire segment since their switching field is reduced to 24 kA/m with respect to the simulated ones (40 kA/m), while the wide segment does not present a significant change. Nanoporous alumina templates typically show a dispersion of the pore diameters, which implies a dispersion on the diameter of the nanowires electrodeposited therein. However, that dispersion is kept constant during the pore widening of the wide segments, which results in a larger relative dispersion of narrow segment diameters, thus showing a wider switching field distribution.

**Figure 6:**
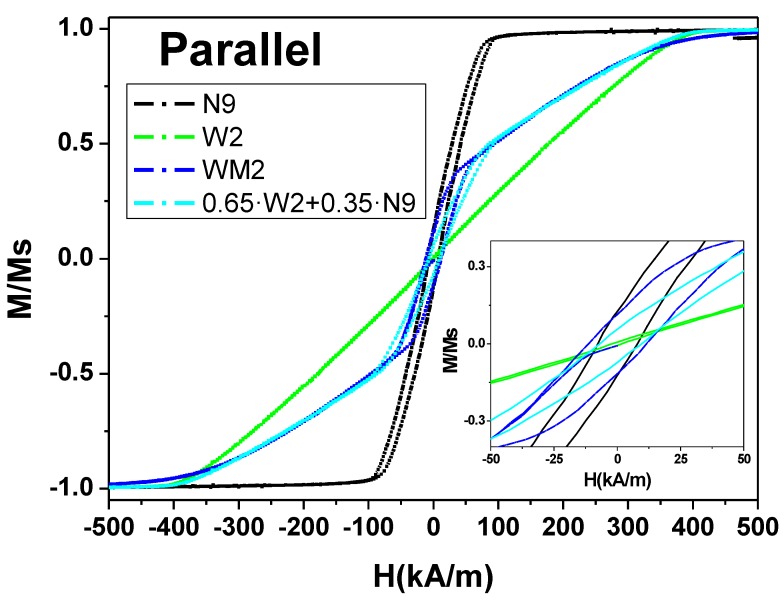
VSM parallel hysteresis loops of nanowire arrays containing narrow segments (N9-black), wide segments (W2-green), narrow and wide segments (WM2-blue), and the weighted average of normalized hysteresis loops of single segment arrays (0.65W2+0.35N9-cyan).

Taking into account the impossibility of performing an exhaustive statistical study with the MOKE system, it is worth employing bulk magnetometry as VSM in order to measure large amounts of nanowires while they are still embedded into the alumina membrane. However, a wide segment of sample WM9 presents a volume, which would produce a magnetization one order of magnitude higher than the narrow one making difficult the differentiation of the contributions from both kinds of segments. For this reason, the segmented nanowire array WM2 sample has been fabricated whose wide segment length is reduced to 2 µm. In order to ensure that this sample still follows the same magnetization reversal mechanism as sample WM9, parallel MOKE hysteresis loops have been measured (Figure 5b). Undoubtedly, the switching field of the wider segment is not appreciably modified by the reduction of the segments length, which allows us to conclude that both samples WM9 and WM2 are comparable. Figure 6 summarizes the bulk hysteresis loops of samples W2, N9, and WM2 measured along the nanowires long axis. First of all, the hysteresis loop of sample N9 corresponds to the typical behaviour of bi-stable magnetic nanowires measured along the easy axis of magnetization under the influence of magnetostatic interactions among nanowires, as inferred from the hysteresis loop tilt along the applied magnetic field axis. However, the coercive field of such interacting nanowire arrays underestimates the mean switching field of the nanowires in a proportion that depends on the interaction strength [53]. The extreme situation can be observed in the hysteresis loop of sample W2 where the strength of magnetostatic interaction is expected to be five times higher as a consequence of the large diameter of the nanowires for the same interpore distance [45,54,55]. Under the effect of the demagnetizing field due to the magnetostatic interactions, the antiparallel coupling of the NW in densely packed arrays would show zero net remanence of magnetization and, thus, anhysteretic behaviour, as evidenced in the hysteresis loop of the W2 sample. Even if single-segment magnetization behaviour cannot be extracted from the bulk hysteresis loops, both samples N9 and W2 show a completely different overall magnetization reversal which can be used to identify the narrow and wide segment contributions to the measured WM2 bulk hysteresis loops. As can be seen in Figure 6, the hysteresis loops of sample WM2 shows two pronounced contributions whose hysteresis can be associated with samples N9 and W2. In fact, the normalized N9 and W2 hysteresis loops have been weight superposed after considering the different contributions to the overall magnetic signal. As a result, the wide segment would show a contribution to the net magnetic moment, around of 65% higher than the narrow one, in order to best fit the experimental hysteresis loop of sample WM2, as shown in Figure 6. As both segments are composed of the same composition, the only fact that determines the different contribution to the magnetic signal is the different volume of each segment. It is worth emphasizing again the impossibility to perform enough statistics on single nanowires, and that also affects the measurement of the segment diameter. From these results, in order to fulfil the 65% larger volume of the wide segment, the mean narrow segment diameter should be closer to 150 nm, although the dispersion of the wide segment diameter, as well as both segment lengths, would affect the relative volume ratio between the segments. Nevertheless, the perfect fitting of the superposed 0.65·W2 + 0.35·N9 hysteresis loop with the experimental WM2 hysteresis loop indicates a representative decoupling between the magnetization switching of each nanowire segments over the whole sample. 

## 4. Conclusions

In summary, by employing a combination of electrochemical anodization, ALD, wet chemical etching, and electrodeposition techniques, it has been possible to produce geometrically-modulated bi-segmented Fe_17_Ni_83_ nanowires with an unprecedented sharp modulation in diameter, ranging from 120 nm for the thinner segment up to 450 nm of the thicker one. These bi-segmented Fe_17_Ni_83_ diameter modulated nanowires display a peculiar two-step magnetization reversal process characterized by two Barkhausen jumps, as demonstrated by the magneto-optical Kerr effect measurements performed in single freestanding nanowires. This experimental finding has been also further supported and explained by micromagnetic simulations performed in this work, which evidence a pinning of the domain wall propagation at the interface of the nanowire diameter modulation. These micromagnetic simulations show good correlation in coercive fields for wider segments (16 kA/m). Furthermore, the difference of 16 kA/m between the simulated and experimental coercive fields of the narrow one can also be explained by taking into account that simulations run a single nanowire with 120 nm diameter, but, experimentally, the mean diameter could be closer to 150 nm. Moreover, from the magnetic hysteresis loops measurements of the bulk nanowires array, it was also possible to conclude that this two-step magnetization reversal might be characteristic of all nanowires in the array, due to the stepwise shape of the magnetic permeability during the magnetization reversal processes of these bi-segmented diameter modulated nanowires. These remarkable results point out to the fact that by properly engineering a sharp geometrical modulation in the diameter of the nanowires, it becomes possible to obtain bi-segmented nanowires with independent magnetization reversal processes for each of different nanowire segments, which constitutes the fundament of novel ultrahigh-density data storage devices based on magnetic multi-domain systems, such as racetrack memories.

## References

[1] Shimanovich D.L., Vorobjova A.I., Tishkevich D.I., Trukhanov A.V, Zdorovets M.V, Kozlovskiy A.L. (2018). Preparation and morphology-dependent wettability of porous alumina membranes. Beilstein J. Nanotechnol..

[2] Porta-i-Batalla M., Xifré-Pérez E., Eckstein C., Ferré-Borrull J., Marsal L.F. (2017). 3D Nanoporous Anodic Alumina Structures for Sustained Drug Release. Nanomaterials.

[3] Martín J., Martín-González M., Francisco Fernández J., Caballero-Calero O. (2014). Ordered three-dimensional interconnected nanoarchitectures in anodic porous alumina. Nat. Commun..

[4] Porta-i-Batalla M., Eckstein C., Xifré-Pérez E., Formentín P., Ferré-Borrull J., Marsal L.F. (2016). Sustained, Controlled and Stimuli-Responsive Drug Release Systems Based on Nanoporous Anodic Alumina with Layer-by-Layer Polyelectrolyte. Nanoscale Res. Lett..

[5] Jeon G., Yang S.Y., Kim J.K. (2012). Functional nanoporous membranes for drug delivery. J. Mater. Chem..

[6] Banerjee P., Perez I., Henn-Lecordier L., Lee S.B., Rubloff G.W. (2009). Nanotubular metal–insulator–metal capacitor arrays for energy storage. Nat. Nanotechnol..

[7] Saranti K., Alotaibi S., Paul S. (2016). A new approach for two-terminal electronic memory devices—Storing information on silicon nanowires. Sci. Rep..

[8] Romero V., Vega V., García J., Prida V.M., Hernando B., Benavente J. (2014). Effect of Porosity and Concentration Polarization on Electrolyte Diffusive Transport Parameters through Ceramic Membranes with Similar Nanopore Size. Nanomaterials.

[9] Kumeria T., Santos A., Rahman M.M., Ferré-Borrull J., Marsal L.F., Losic D. (2014). Advanced Structural Engineering of Nanoporous Photonic Structures: Tailoring Nanopore Architecture to Enhance Sensing Properties. ACS Photonics.

[10] Santos A., Kumeria T., Wang Y., Losic D. (2014). In situ monitored engineering of inverted nanoporous anodic alumina funnels: On the precise generation of 3D optical nanostructures. Nanoscale.

[11] Hanawa T. (2009). Materials for metallic stents. J. Artif. Organs.

[12] Swan E.E.L., Popat K.C., Grimes C.A., Desai T.A. (2005). Fabrication and evaluation of nanoporous alumina membranes for osteoblast culture. J. Biomed. Mater. Res. Part A.

[13] Osmanbeyoglu H.U., Hur T.B., Kim H.K. (2009). Thin alumina nanoporous membranes for similar size biomolecule separation. J. Memb. Sci..

[14] Fernández-Pacheco A., Streubel R., Fruchart O., Hertel R., Fischer P., Cowburn R.P. (2017). Three-dimensional nanomagnetism. Nat. Commun..

[15] Coïsson M., Celegato F., Barrera G., Conta G., Magni A., Tiberto P. (2017). Bi-Component Nanostructured Arrays of Co Dots Embedded in Ni_80_Fe_20_ Antidot Matrix: Synthesis by Self-Assembling of Polystyrene Nanospheres and Magnetic Properties. Nanomaterials.

[16] Sander D., Valenzuela S.O., Makarov D., Marrows C.H., Fullerton E.E., Fischer P., McCord J., Vavassori P., Mangin S., Pirro P. (2017). The 2017 Magnetism Roadmap. J. Phys. D Appl. Phys..

[17] Chen Y., Xu C., Zhou Y., Maaz K., Yao H., Mo D., Lyu S., Duan J., Liu J. (2016). Temperature- and Angle-Dependent Magnetic Properties of Ni Nanotube Arrays Fabricated by Electrodeposition in Polycarbonate Templates. Nanomaterials.

[18] Abad B., Maiz J., Ruiz-Clavijo A., Caballero-Calero O., Martin-Gonzalez M. (2016). Tailoring thermal conductivity via three-dimensional porous alumina. Sci. Rep..

[19] Lavrijsen R., Lee J.-H., Fernández-Pacheco A., Petit D.C.M.C., Mansell R., Cowburn R.P. (2013). Magnetic ratchet for three-dimensional spintronic memory and logic. Nature.

[20] Wagner M.F.P., Völklein F., Reith H., Trautmann C., Toimil-Molares M.-E. (2016). Fabrication and thermoelectrical characterization of three-dimensional nanowire networks. Phys. Status Solidi.

[21] Van Thiem L., Tu L.T., Phan M.-H. (2015). Magnetization Reversal and Magnetic Anisotropy in Ordered CoNiP Nanowire Arrays: Effects of Wire Diameter. Sensors.

[22] Caballero-Calero O., Martín-González M. (2016). Thermoelectric nanowires: A brief prospective. Scr. Mater..

[23] Bochmann S., Fernandez-Pacheco A., Mačković M., Neff A., Siefermann K.R., Spiecker E., Cowburn R.P., Bachmann J. (2017). Systematic tuning of segmented magnetic nanowires into three-dimensional arrays of ‘bits’. RSC Adv..

[24] Cisternas E., Vogel E.E. (2013). Inscription and stabilization of ferromagnetic patterns on arrays of magnetic nanocylinders. J. Magn. Magn. Mater..

[25] Cisternas E., Vogel E.E. (2015). Improving information storage by means of segmented magnetic nanowires. J. Magn. Magn. Mater..

[26] Cisternas E., Faúndez J., Vogel E.E. (2017). Stabilization mechanisms for information stored in magnetic nanowire arrays. J. Magn. Magn. Mater..

[27] Prida V.M., García J., Iglesias L., Vega V., Görlitz D., Nielsch K., Barriga-Castro E.D., Mendoza-Reséndez R., Ponce A., Luna C. (2013). Electroplating and magnetostructural characterization of multisegmented Co_54_Ni_46_/Co_85_Ni_15_ nanowires from single electrochemical bath in anodic alumina templates. Nanoscale Res. Lett..

[28] Méndez M., González S., Vega V., Teixeira M.J., Hernando B., Luna C., Prida M.V. (2017). Ni-Co Alloy and Multisegmented Ni/Co Nanowire Arrays Modulated in Composition: Structural Characterization and Magnetic Properties. Crystals.

[29] Palmero E.M., Béron F., Bran C., del Real R.P., Vázquez M. (2016). Magnetic interactions in compositionally modulated nanowire arrays. Nanotechnology.

[30] Ivanov Y.P., Chuvilin A., Lopatin S., Kosel J. (2016). Modulated Magnetic Nanowires for Controlling Domain Wall Motion: Toward 3D Magnetic Memories. ACS Nano.

[31] Trukhanov A.V, Grabchikov S.S., Vasiliev A.N., Sharko S.A., Mukhurov N.I., Gasenkova I. (2014). V Specific features of formation and growth mechanism of multilayered quasi-one-dimensional (Co-Ni-Fe)/Cu systems in pores of anodic alumina matrices. Crystallogr. Rep..

[32] Trukhanov A.V, Grabchikov S.S., Sharko S.A., Trukhanov S.V, Trukhanova K.L., Volkova O.S., Shakin A. (2016). Magnetotransport properties and calculation of the stability of GMR coefficients in CoNi/Cu multilayer quasi-one-dimensional structures. Mater. Res. Express.

[33] Rodríguez L.A., Bran C., Reyes D., Berganza E., Vázquez M., Gatel C., Snoeck E., Asenjo A. (2016). Quantitative Nanoscale Magnetic Study of Isolated Diameter-Modulated FeCoCu Nanowires. ACS Nano.

[34] Salem M.S., Tejo F., Zierold R., Sergelius P., Moreno J.M.M., Goerlitz D., Nielsch K., Escrig J. (2018). Composition and diameter modulation of magnetic nanowire arrays fabricated by a novel approach. Nanotechnology.

[35] Bran C., Berganza E., Fernandez-Roldan J.A., Palmero E.M., Meier J., Calle E., Jaafar M., Foerster M., Aballe L., Fraile Rodriguez A. (2018). Magnetization Ratchet in Cylindrical Nanowires. ACS Nano.

[36] Grabchikov S.S., Trukhanov A.V., Trukhanov S.V., Kazakevich I.S., Solobay A.A., Erofeenko V.T., Vasilenkov N.A., Volkova O.S., Shakin A. (2016). Effectiveness of the magnetostatic shielding by the cylindrical shells. J. Magn. Magn. Mater..

[37] Wai P., Dmitrenko V., Grabchikov S., Vlasik K., Novikov A., Petrenko D., Trukhanov V., Ulin S., Uteshev Z., Chernysheva V. (2016). Application peculiarities of magnetic materials for protection from magnetic fields. J. Phys. Conf. Ser..

[38] Trukhanov A.V., Grabchikov S.S., Solobai A.A., Tishkevich D.I., Trukhanov S.V., Trukhanova E.L. (2017). AC and DC-shielding properties for the Ni80Fe20/Cu film structures. J. Magn. Magn. Mater..

[39] Prida V.M., Salaheldeen M., Pfitzer G., Hidalgo A., Vega V., González S., Teixeira J.M., Fernández A., Hernando B. (2017). Template Assisted Deposition of Ferromagnetic Nanostructures: From Antidot Thin Films to Multisegmented Nanowires. Acta Phys. Pol. A.

[40] Nielsch K., Choi J., Schwirn K., Wehrspohn R.B., Gösele U. (2002). Self-ordering Regimes of Porous Alumina:  The 10 Porosity Rule. Nano Lett..

[41] Proenca M.P., Sousa C.T., Leitao D.C., Ventura J., Sousa J.B., Araujo J.P. (2008). Nanopore formation and growth in phosphoric acid Al anodization. J. Non. Cryst. Solids.

[42] Bachmann J., Zierold R., Chong Y.T., Hauert R., Sturm C., Schmidt-Grund R., Rheinländer B., Grundmann M., Gösele U., Nielsch K. (2008). A Practical, Self-Catalytic, Atomic Layer Deposition of Silicon Dioxide. Angew. Chemie. Int. Ed..

[43] Zierold R., Wu Z., Biskupek J., Kaiser U., Bachmann J., Krill C.E., Nielsch K. (2010). Magnetic, Multilayered Nanotubes of Low Aspect Ratios for Liquid Suspensions. Adv. Funct. Mater..

[44] Vega V., Böhnert T., Martens S., Waleczek M., Montero-Moreno J.M., Görlitz D., Prida V.M., Nielsch K. (2012). Tuning the magnetic anisotropy of Co–Ni nanowires: Comparison between single nanowires and nanowire arrays in hard-anodic aluminum oxide membranes. Nanotechnology.

[45] Raposo V., Zazo M., Flores A.G., Garcia J., Vega V., Iñiguez J., Prida V.M. (2016). Ferromagnetic resonance in low interacting permalloy nanowire arrays. J. Appl. Phys..

[46] Vansteenkiste A., Leliaert J., Dvornik M., Helsen M., Garcia-Sanchez F., Van Waeyenberge B. (2014). The design and verification of MuMax3. AIP Adv..

[47] Zhao Y., Song Q., Yang S.-H., Su T., Yuan W., Parkin S.S.P., Shi J., Han W. (2016). Experimental Investigation of Temperature-Dependent Gilbert Damping in Permalloy Thin Films. Sci. Rep..

[48] Salem M.S., Sergelius P., Corona R.M., Escrig J., Gorlitz D., Nielsch K. (2013). Magnetic properties of cylindrical diameter modulated Ni_80_Fe_20_ nanowires: Interaction and coercive fields. Nanoscale.

[49] Rodríguez L.A., Deen L., Córdoba R., Magén C., Snoeck E., Koopmans B., De Teresa J.M. (2015). Influence of the shape and surface oxidation in the magnetization reversal of thin iron nanowires grown by focused electron beam induced deposition. Beilstein J. Nanotechnol..

[50] Wieser R., Nowak U., Usadel K.D. (2003). Domain wall mobility in nanowires: Transverse versus vortex walls. Phys. Rev. B.

[51] Bran C., Fernandez-Roldan J.A., Palmero E.M., Berganza E., Guzman J., del Real R.P., Asenjo A., Rodriguez A.F., Foerster M., Aballe L. (2017). Direct Observation of Transverse and Vortex Metastable Magnetic Domains observed in Cylindrical Nanowires. Phys. Rev. B.

[52] Fernandez-Roldan J., Perez del Real R., Bran C., Vazquez M., Chubykalo-Fesenko O. (2018). Magnetization pinning in modulated nanowires: From topological protection to the “corkscrew” mechanism. Nanoscale.

[53] Sergelius P., Fernandez J.G., Martens S., Zocher M., Böhnert T., Martinez V.V., de la Prida V.M., Görlitz D., Nielsch K. (2016). Statistical magnetometry on isolated NiCo nanowires and nanowire arrays: A comparative study. J. Phys. D Appl. Phys..

[54] Encinas-Oropesa A., Demand M., Piraux L., Huynen I., Ebels U. (2001). Dipolar interactions in arrays of nickel nanowires studied by ferromagnetic resonance. Phys. Rev. B.

[55] Vega V., Prida V.M., García J.A., Vazquez M. (2010). Torque magnetometry analysis of magnetic anisotropy distribution in Ni nanowire arrays. Phys. Status Solidi.

